# Elevated EGFL6 modulates cell metastasis and growth via AKT pathway in nasopharyngeal carcinoma

**DOI:** 10.1002/cam4.1883

**Published:** 2018-11-15

**Authors:** Ziyu Zhu, Haosheng Ni, Bo You, Si Shi, Ying Shan, Lili Bao, Bingyue Duan, Yiwen You

**Affiliations:** ^1^ Department of Otorhinolaryngology Head and Neck surgery Affiliated Hospital of Nantong University Nantong China

**Keywords:** AKT, EGFL6, growth, metastasis, nasopharyngeal carcinoma

## Abstract

Epidermal growth factor‐like domain multiple 6 (EGFL6) is a secreted protein, regulates maintenance and metastasis of cancer cells. Nevertheless, how EGFL6 participates in the progression and tumorigenesis of nasopharyngeal carcinoma (NPC) remains unclear. In our study, EGFL6 was detected highly expressed in 20 NPC tissues compared with normal tissues by IHC assay. Then, the level of EGFL6 in NPC serum and NPC cells was explored through enzyme‐linked immunosorbent assay and western blot, the results consistent with IHC. More interestingly, EGFL6 accelerated the migration and growth of NPC in vitro assays. Considering the mechanism of migration, NPC cells were cultured with AKT activator, revealing EGFL6 facilitated the progression of NPC via AKT. Moreover, the same effect of EGFL6 in promoting NPC growth was proved in nude mice. Furthermore, heat‐shock zebrafish model was established with EGFL6 overexpression. Then, CNE2 cells were injected into the model and cells mass was observed, showing that EGFL6 enhanced the migration and metastasis of NPC. Currently, as the prognosis of NPC is severely affected by distant metastasis, it might be a new therapeutic target toward EGFL6. Taken together, our results suggested that EGFL6 acts as a potential positive regulator in the migration and proliferation of NPC.

## 1 INTRODUCTION

Nasopharyngeal carcinoma (NPC) is a squamous epithelial cell carcinoma with an incidence of 15‐50 cases per 100 000 and is especially prevalent in Southeast Asia.^1^, ^2^ Currently, with the application of intensity‐modulated radiotherapy, the locoregional control rate of NPC is much higher than before.^3^ But the prognosis of NPC patients still remains poor because of distant metastasis and recurrence.^4^ Thus, it is of great clinical value to further explore the underlying molecular mechanisms of proliferation and migration of NPC.

Epidermal growth factor‐like domain multiple 6 (EGFL6) is an extracellular matrix protein, belongs to EGFL family and plays an important role in promoting the migration and angiogenesis of endothelial cells.^5^, ^6^ Recently, it is reported that EGFL6 regulates the metastasis of ALDH^+^ ovarian cancer cells and is overexpression in benign meningioma and oral squamous cell carcinoma.^5^, ^7^, ^8^ Moreover, EGFL6 implicates in the pathogenesis of benign meningioma via activating AKT pathway.^9^ However, there is some lack of knowledge about the expression and the relationship of EGFL6 in the function and mechanisms of NPC.

In present study, we first found EGFL6 is upregulated in NPC (tissues, serum and cells). To further explore the function of EGFL6 in NPC, we silenced EGFL6 by EGFL6‐RNAi (shEGFL6) in vitro. The result revealed that EGFL6 is positively correlated with proliferation and migration of NPC. The activation of AKT is necessary for several functions of cancer cells, including apoptosis, migration, invasion, and angiogenesis.^10^, ^11^, ^12^, ^13^ Subsequently, we observed that EGFL6 increased the migration by AKT signaling pathway in NPC. Furthermore, we demonstrated the mediation role of EGFL6 in nude mice. Furthermore, we established the heat‐shock zebrafish model with EGFL6 overexpression. The metastatic ability of NPC was evaluated via counting the cell mass in zebrafish model.

## 2 MATERIALS AND METHODS

### 2.1 Specimens collection and ethics statement

Thirty‐one NPC tissues and serum were obtained from the Affiliated Hospital of Nantong University, China. Fifteen non‐cancerous nasopharyngeal (N) tissues and serum were chronic inflammatory nasopharyngeal epithelium tissues and serum, collected from those patients who were suspected to have NPC, but ruled out by the diagnosis of pathological. All the clinical experiments were performed with the consent of patients and obtained the approval from the Ethics Committee of Affiliated Hospital of Nantong University.

### 2.2 Immunohistochemistry (IHC)

We rehydrated the paraffin‐embedded NPC and N tissues, and incubated the sections with citrate buffer for antigen retrieval and heated in an autoclave. Subsequently, we performed the immunohistochemistry with DAB Detection Kit (ZSGB, Beijing, China) following the instructions of manufacturer and counterstained the sections with hematoxylin. The sections were incubated with primary antibody: anti‐EGFL6 antibody (1:100, ab140079, Abcam, MA, USA).

The expression level of EGFL6 was scored by the staining intensity and distribution. The intensity of EGFL6 was scored as four grades: 0, no staining, 1, weak, 2, moderate, and 3, strong. The percentage of positive cells were graded as 0 (0%), 1 (1%‐33%), 2 (34%‐66%), and 3 (67%‐100%). The final evaluation of staining score was according to the product of the intensity and percentage scores.

### 2.3 Cell culture

All four nasopharyngeal carcinoma cell lines CNE1, CNE2, 5‐8F, and 6‐10B were maintained in RPMI medium 1640 (GibCo BRL, Grand Island, NY) supplemented with 10% FBS (Gibco). The immortalized normal nasopharyngeal epithelial cell line NP69 was grown in Keratinocyte‐serum‐free medium (Invitrogen, Carlsbad, CA). All the cells were maintained at 37°C and in 5% CO_2_ in Otolaryngology Laboratory, Affiliated Hospital of Nantong University.

### 2.4 Plasmid transfection

The EGFL6‐RNAi (shEGFL6) was designed and obtained from Genechem (Shanghai, China) (GIEE0125761). The mock GV102 vector was used as the negative control (shNC). NPC cells were grown in six‐well plates and transfected with 2 μg of DNA plasmids with Lipofectamine 2000 (Invitrogen) according to the manufacturer's instructions. After incubated for 48 hours, we observed the fluorescence of the transfection efficiency by inverted microscope (Zeiss, Gottingen, Germany).

### 2.5 Western blot analysis

The transfected cells were lysed using RIPA Lysis Buffer containing protease inhibitor PMSF on ice. We measured the protein concentration by the BCA Protein Assay Kit (Thermo Scientific, Waltham, MA, USA). About 20 μg protein was separated by (SDS‐PAGE) and transferred onto PVDF membranes (Millipore, Billerica, MA). The membranes were blocked in 5% non‐fat dry milk with Tris‐buffered saline (TBST) for 1 hour at room temperature, and then incubated the membranes with primary antibody overnight at 4°C, after that they were incubated with HRP‐tagged secondary antibody (1:3000; Sangon Biotech, Shanghai, China) for 1 hour at room temperature. Then, we detected the immunoreactivity by enhanced chemiluminescence system (ECL, Cell Signaling Technologies, Beverly, MA, USA). Image J software was used for the densitometry analysis of each group, *β*‐actin was used as a loading control. We used the following antibodies: anti‐EGFL6 primary antibody (1:1000, ab140079, Abcam), anti‐β‐actin (1:1000, D110001‐0100, Sangon Biotech), anti‐p‐AKT (1:1000, Ser473, CST), anti‐AKT (1:1000, C67E7, CST). All the experiments were repeated three times.

### 2.6 Transwell assay

Nasopharyngeal carcinoma cells were transfected with shEGFL6 and shNC, and 48 hours later 50 000 cells were trypsinized and reseeded with 200 μL serum‐free medium on the top chambers of 24‐well transwell culture inserts (8‐μm pore size, coring, China). After incubated for 17 hours, the cells that had migrated to the undersurface of the membrane were fixed in 4% paraformaldehyde for 30 minutes, and then stained with 0.05% crystal violet for 30 minutes. Subsequently, three random fields were counted and captured under the inverted microscope (Zeiss). All the experiments were repeated for three times.

### 2.7 Quantitative analysis of blood serum EGFL6 level

The concentration of EGFL6 in NPC serum samples was estimated quantitatively by the enzyme‐linked immunosorbent assay (ELISA) following the manufacturer’ instructions (Human EGFL6 ELISA Kit, EL007475HU, CUSABIO). About 100 μL standard control sample and plasma sample were added into the microtiter plates coated with EGFL6 antibody and incubated for 2 hours at 37°C. The absorbance was measured at 450 nm, and the data were analyzed by *Curve Expert*.

### 2.8 Cell cycle analyses

The transfected NPC cells were trypsinized and harvested after 72 hours. Subsequently, we fixed the cells in 70% ethanol at −20°C for 24 hours. Then, the cells were incubated with 1% TritonX‐100 10 minutes, and stained with PI/RNase Staining Buffer 20 minutes (BD Biosciences, San Diego, CA). After all, the samples were analyzed by FACS Calibur Flow Cytometer (BD Bioscience) and BD CellQuest software (BD Bioscience).

### 2.9 Immunofluorescence assay

We harvested the transfected NPC cells and reseeded to 0.8 cm×0.8 cm glass coverslips overnight in the 24‐well plate. Then, 4% paraformaldehyde was used to fixed and washed in PBS. After that, the cells were incubated with anti‐Ki67 antibody (1:50, 27309‐1‐AP, Proteintech, Wuhan, China) overnight. PBS washed and incubated with Cy3 (1:100, SA00009‐2, Proteintech, Wuhan, China) labeled secondary antibody. And the nuclei were stained with Hochest. Then, the cells were observed under the Zeiss microscope.

### 2.10 Quantitative real‐time PCR (qRT‐PCR) analysis

The transfection efficiency of shEGFL6 was measured by SYBR qRT‐PCR. We extracted total RNA of the transfected cells with TRIzol (Invitrogen) following the manufacturer's instructions. Then, the cDNA was synthesized from 4 μg of total RNA. The primers we detected EGFL6 mRNA expression by quantitative PCR were as follows: EGFL6 forward primer: 5′‐ATGTTACCCCAGAACCCACC‐3′, reverse primer: 5′‐AGGCTTCGCTCCTCTATGTC‐3′, GAPDH forward: 5′‐CAGGAGGCATTGCTGATGAT‐3′, reverse primer 5′‐GAAGGCTGGGGCTCATTT‐3′, they were designed by BioSune Co., Ltd (Shanghai, China). About 20 μL reactions containing 0.4 μL forward primer, 0.4 μL reverse primer, 1 μL cDNA, 10 μL SYBR (sigma, Mannheim, Germany), and 8.2 μL DEPC water were subjected to 1 cycle at 95°C followed by 40 cycles of 95°C for 2 seconds, 60°C for 30 seconds, and 72°C for 10 seconds. GAPDH was used to normalize the expression of EGFL6.

### 2.11 Animal xenograft tumor model

All experiments were performed with institutional guidelines and approved by the committee on the Ethics of Animal Experiments of Nantong University. About 1 × 10^6^ CNE2 cells with transfection were injected into BALB/C‐Nu nude mice (5 weeks old) (Shanghai Laboratory Animal Center, China) for subcutaneous injection. We measured the length and width of the visible tumor every two days, and the volume of tumor was calculated as (length × width2)/2. After 12 days, all the mice were sacrificed and the final tumor volumes were calculated.

### 2.12 Heat‐shock zebrafish metastatic model

#### 2.12.1 Zebrafish strains and husbandry

Zebrafish strains and husbandry zebrafish embryos, larvae, and adult fish were raised in the Zebrafish Center of Nantong University. The experiment was approved based in Animal Protection Laws of China. Zebrafish lines used in this study were AB wildtype and fli1ep: CAAX‐EGFP strain. Microinjection was performed when embryos are at 1‐2‐cell stage via a microinjection system (WPI).

#### 2.12.2 Vector construction

All Tol2‐flanked vectors were generated by inserting transgenic components into a Tol2 destination vector, using multisite gateway clonings. All entry and expression constructs were generated via LR reaction, in which attL and attR sites recombine, as described.^14^ Donor vectors, hsp70l promoter, polyA were adopted from a multisite gateway‐based construction kit for Tol2 transposon transgenesis constructs, and (pME: EGFL6‐EGFP) were synthetized by biologic company. For heat‐shocked procedures, the injected embryos were bathed in pre‐heated E3 media at 37°C for 1 hour.

#### 2.12.3 Metastatic model

Zebrafish metastatic model was used to evaluate the metastatic of NPC.^15^ In our study, CNE2 cells were firstly stained with 2 g/mL of DiI (Fluka, Germany) for 30 minutes. After that, 100‐500/5 nL CNE2 cells were counted to resuspend in serum‐free 1640 medium and injected into embryos at 48 hpf. All images were taken from whole‐mount samples, including bright‐field and fluorescent channels, via a fluorescent microscope (Leica).

### 2.13 Statistical analysis

All the experiments were performed at least in triplicate. Results were displayed as mean ± SD. Statistical analysis was performed by GraphPad Prism^®^ version 5.01. Student's *t* test was analyzed to determine the significance of differences in groups. *P* < 0.05 was considered to be statistically significant.

## 3 RESULTS

### 3.1 The expression of EGFL6 in NPC

**Figure 1:**
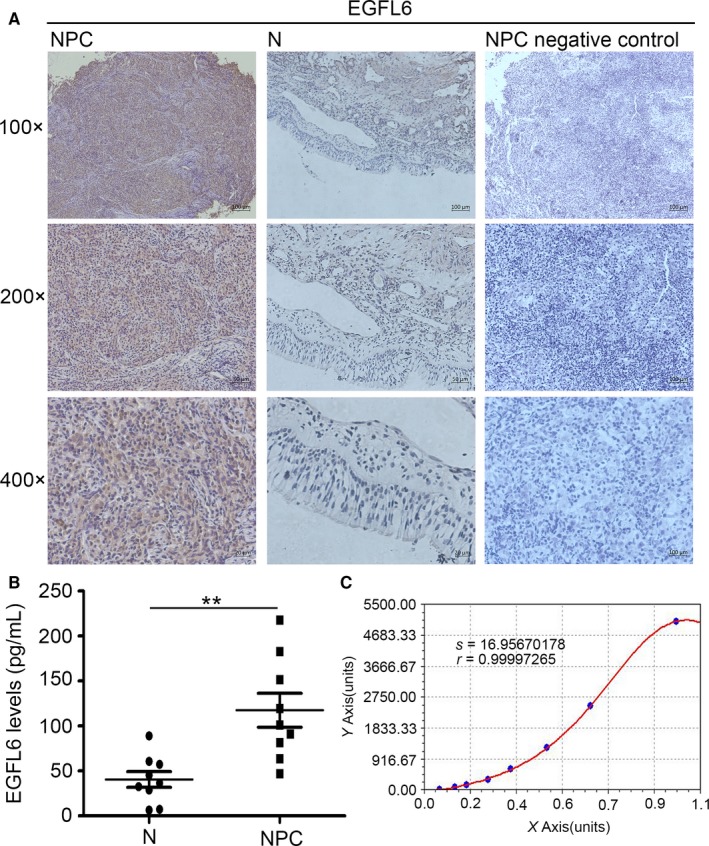
The expression of EGFL6 in nasopharyngeal carcinoma (NPC). A, High expression of EGFL6 was observed in NPC tissues compared with non‐cancerous nasopharyngeal (N) tissues by immunohistochemistry analysis. B, High level of EGFL6 in NPC serum compared with N serum. **P < 0.01. C, The standard curve analysis of EGFL6 in serum

We first detected the expression of EGFL6 in non‐cancerous nasopharyngeal tissues (N) and nasopharyngeal carcinoma (NPC). As shown in Figure 1A, the positive expression lever of EGFL6 in NPC was much higher than in N. As EGFL6 is a secretory protein, the level of it was also detected in NPC samples blood serum by ELISA kit. We found the higher level of EGFL6 in NPC blood serum (Figure 1B), basing the calculation of sample values from the *Curve Expert* (Figure 1C). These data suggested that the level of EGFL6 is elevated in NPC tissues and blood serum.

### 3.2 EGFL6 promoted migration and proliferation of NPC cells

**Figure 2:**
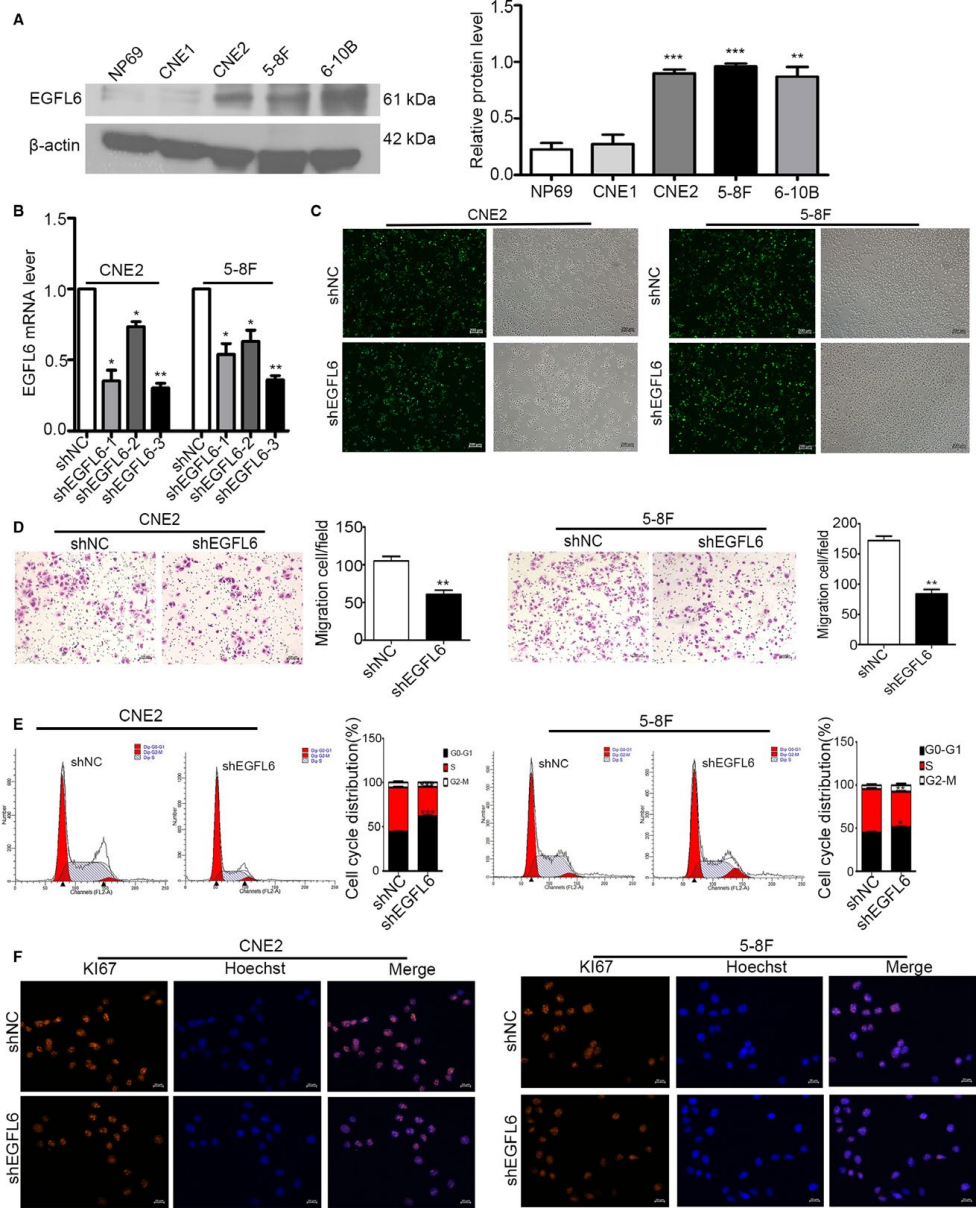
EGFL6 promoted the migration and proliferation of nasopharyngeal carcinoma (NPC) cells. A, Western blot analysis of the expression of EGFL6 in NPC cell lines and normal nasopharyngeal epithelial (NP69) cells. Actin was used as a loading control. Data are the means ± SD. **P < 0.01, ***P < 0.001. B, CNE2 and 5‐8F cells were transfected with shEGFL6 (1, 2, 3) and shNC. 48 hours later, total RNA was extracted from those cells and the levels of EGFL6 were measured by qRT‐PCR. *P < 0.05, **P < 0.01. C, The fluorescent transfection efficiency of NPC cells was captured by inverted microscope. D, The effect of EGFL6 on migration of NPC cells was observed by transwell. **P < 0.01. E, Flow cytometry was performed to quantify the progress of cell cycle, *P < 0.05, **P < 0.01, ***P < 0.001. F, Immunofluorescence analysis of KI67 expression in NPC cells of shEGFL6 and shNC group. The nucleus was counterstained with Hoechst in blue. KI67 staining was in red

To explore the biological roles of EGFL6 in NPC further, we carried out western blot to evaluate the expression of EGFL6 in four NPC cell lines (CNE1, CNE2, 5‐8F, and 6‐10B) and a normal nasopharyngeal epithelial cell line (NP69). And, we found the expression of CNE2 cells and 5‐8F cells was remarkably increased compared with NP69 (Figure 2A). Based on this, we chosen CNE2 and 5‐8Fcells for further experiments. We performed EGFL6 knockdown in CNE2 and 5‐8F cells to investigate to the role of EGFL6 in NPC. EGFL6‐RNAi(1, 2, 3) and null vector (shNC) were transfected to NPC cells. Then, we extracted RNA from the transfected cells, and selected EGFL6‐RNAi‐3 (shEGFL6) as the most efficient RhoB‐RNAi for further study (Figure 2B). The transfection efficiency was observed by the fluorescence (Figure 2C).

To determine whether EGFL6 regulates the biological function of NPC, we performed transwell, cell cycle experiment and immunofluorescence in vitro. After transfected shEGFL6 and shNC 48 hours, we collected 50 000 NPC cells to each transwell chamber. Then, we counted the migration cells 18 hours later. As shown in Figure 2D, the migrated cell number in shEGFL6 group decreased obviously both CNE2 and 5‐8F cells. Next, cell cycle experiment was performed to test the effect of EGFL6 in proliferation. Interestingly, we found an increased number in G0‐G1 phase and a decreased number in S phase in shEGFL6 group compared with the shNC group (Figure 2E). It suggested that EGFL6 promotes the proliferation of NPC cells. Furthermore, immunofluorescence microscopy substantiated the results (Figure 2F). We stained NPC cells, transfected with shEGFL6 and shNC, with KI67, which was a classical maker for proliferation. The immunofluorescence staining in shEGFL6 group was much decreased. Taken together, EGFL6 might stimulate the migration and proliferation of NPC cells.

### 3.3 EGFL6 mediated the axis on migration of AKT signaling

**Figure 3:**
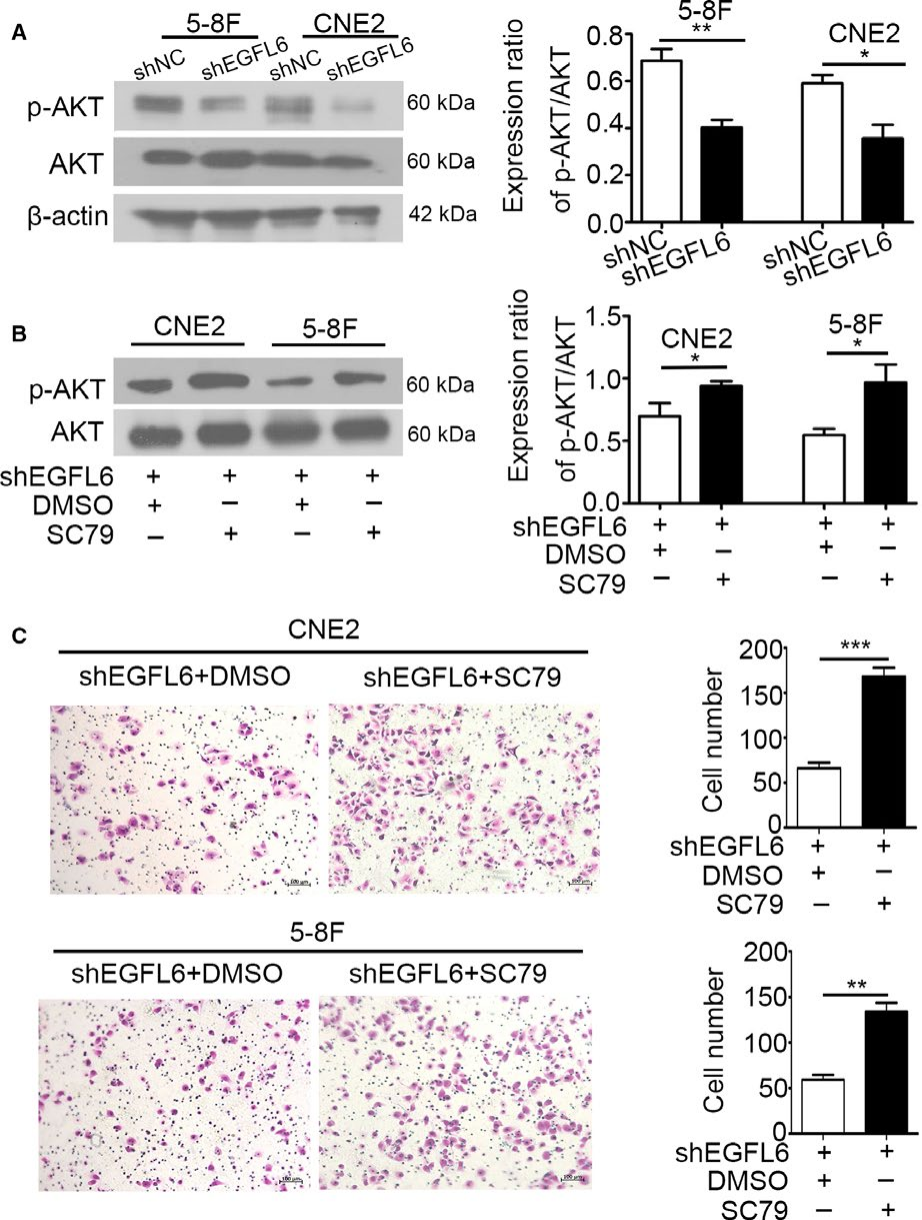
EGFL6 mediated the axis on migration of AKT signaling. A, P‐AKT and AKT in NPC cells transfected with shEGFL6 and shNC was measured by Western blot analysis. β‐actin was used as a loading control. *P < 0.05, **P < 0.01. B, After treated with SC79 or DMSO, the level of p‐AKT and AKT in shEGFL6 NPC cells was detected by Western blot analysis. *P < 0.05. C, ShEGFL6 NPC cells treated with SC79 or DMSO were subjected to transwell assay. **P < 0.01, ***P < 0.001

Considering the mediation of AKT path in the progression of cancers, we also found that p‐AKT was downregulated in shEGFL6 group (Figure 3A). Next, we treated shEGFL6 NPC cells with specific AKT activator SC79 or DMSO for 48 hours, the level of p‐AKT in different groups was shown in Figure 3B. And the NPC cells in different groups were subjected to transwell assay. Interestingly, the migration number of NPC cells was prominently increased after activation of AKT (Figure 3C). Taken together, these phenomena demonstrated that AKT activation is involved in EGFL6 promoting the migration of NPC cells.

### 3.4 The silence of EGFL6 reduced the growth of NPC in nude mice

**Figure 4:**
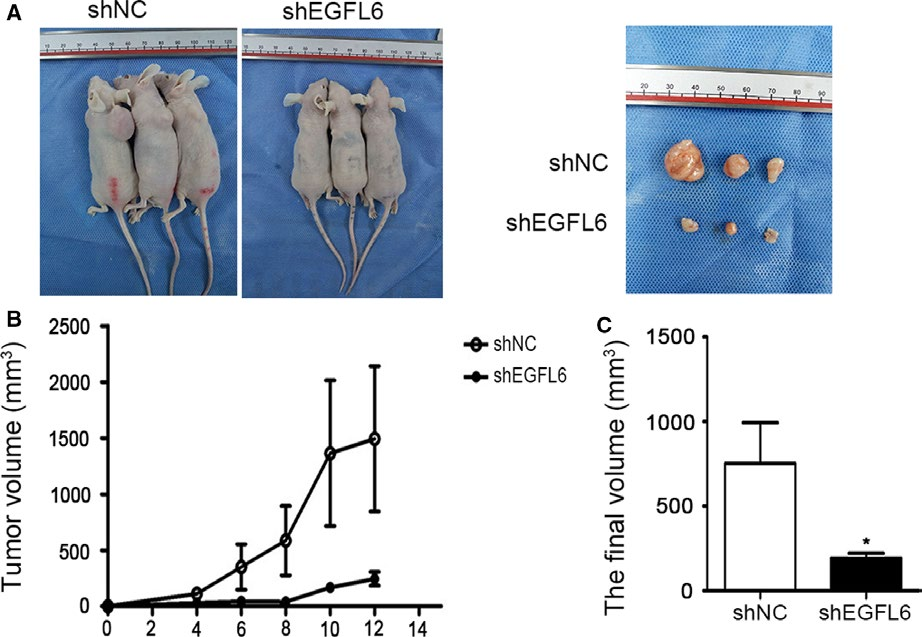
The silence of EGFL6 reduced the growth of NPC in nude mice. A, Representative examples of nude mice subcutaneous injection of CNE2 cells. B, Tumor volumes were evaluated every two days. C, The final tumor volume of sacrificed mice at day 12 was obtained. Mean ± SEM, *P < 0.05

Subsequently, we went on to examine the impact of EGFL6 on NPC progression in vivo. Xenografts model were established by subcutaneous injection of NPC cells, transfected with shEGFL6 and shNC, into nude mice. The tumor size was measured and recorded every 2 days. Consistent with the results in vitro, the tumor volumes in xenografts with silenced EGFL6 were reduced remarkably, compared with control (Figure 4A‐D). These results demonstrated that EGFL6 promoted NPC growth.

### 3.5 EGFL6 gain of function increased the migration of NPC cells in zebrafish

**Figure 5:**
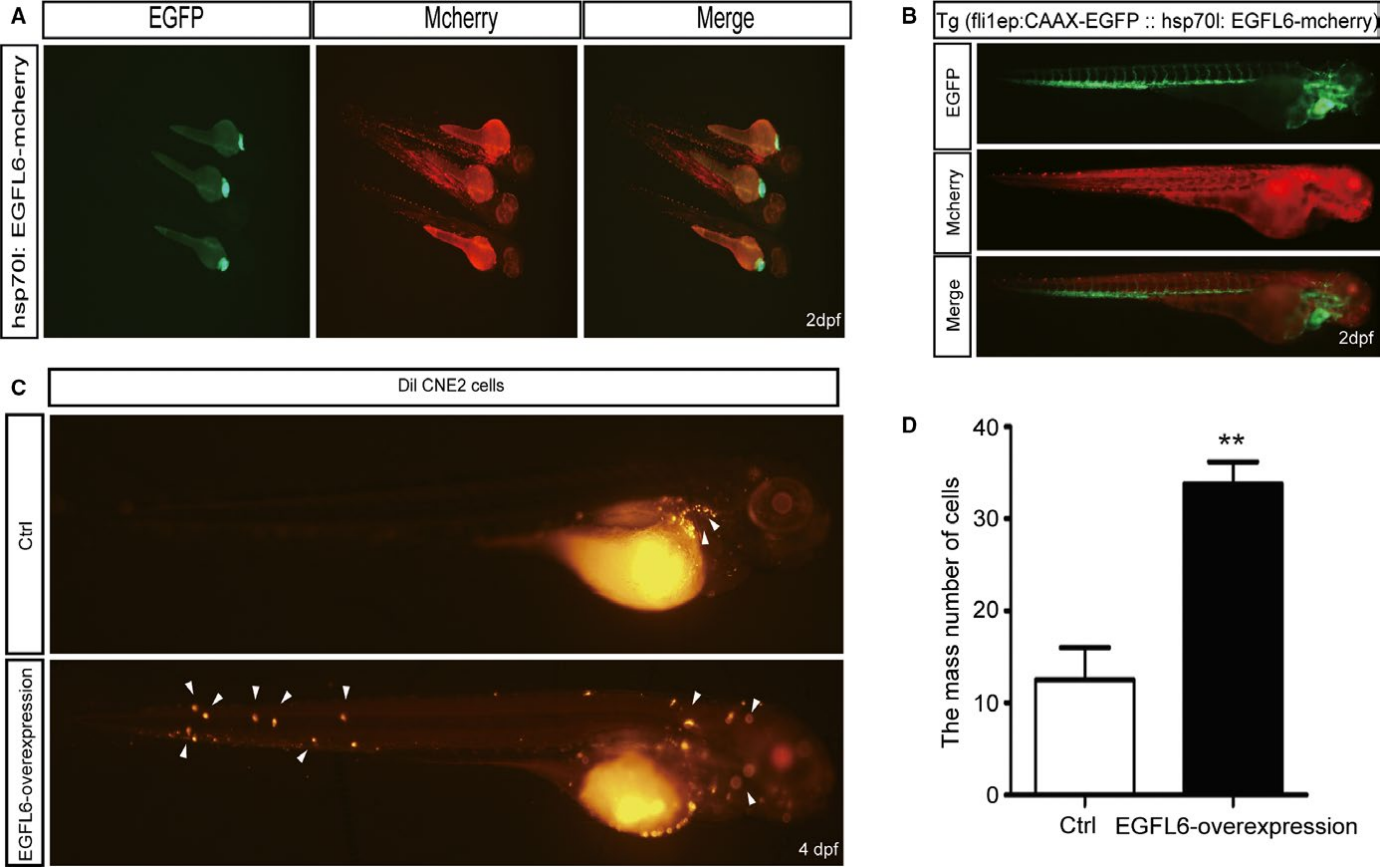
EGFL6 gain of function increased the migration of NPC cells in zebrafish. We selected embryos at 24 h for green hearts, and observed the red fluorescence in embryos after heat‐shocked for 1 h. A, hsp70l:EGFL6‐mcherry embryos at 48 hpf (hours post fertilization), after heat‐shocked for 1 h at 24 hpf. B, hsp70l:EGFL6‐mcherry :: fli1ep: CAAX‐EGFP transgenic line embryos at 48 hours, after heat‐shocked for 1 h at 24 hpf. C and D, CNE2 cells labeled with dil were injected into hsp70l:EGFL6‐mcherry :: fli1ep: CAAX‐EGFP transgenic line embryos at 48 hpf. Then, the migration of CNE2 cells was detected at 2 d post injection. Dpf (days post fertilization). Arrows indicate tumor foci. **P < 0.01

To evaluate the effect of EGFL6 on migration in vivo, we established a heat‐shocked zebrafish, hsp70 l:EGFL6‐mcherry, which upregulated the expression of EGFL6 with bright red fluorescence after heat‐shocked (Figure 5A). Subsequently, we selected embryos with green hearts from hsp70 l:EGFL6‐mcherry:: fli1ep: CAAX‐EGFP, and treated them with heat‐shock. Then, the embryos with red fluorescence were picked up, which upregulated EGFL6 stably (Figure 5B). Next, dil‐labeled CNE2 cells were injected into 48 hours embryos, including EGFL6‐overexpression and ctrl groups. Two days later, the number of migration masses among trunk and head was obviously increased in EGFL6‐overexpression group compared with ctrl (Figure 5C,D). Taken together, EGFL6 promotes the migration of NPC cells in zebrafish.

## 4 DISCUSSION

NPC is notorious for its highly invasive malignancy among head and neck malignancies, metastasis and recurrence have closely association with NPC progression.^16^ Therefore, it becomes more and more important to explore the mechanisms of metastasis and proliferation in NPC. Recently, it has been reported that oncogenes activation and tumor suppressor genes inactivation may be involved in the heterogeneity of tumors.^17^, ^18^ In our study, we found EGFL6 was upregulated in NPC clinical samples tissues, blood serum (Figure 1A‐C), and cell lines (Figure 2A). Furthermore, our findings suggested that EGFL6 could promote the migration and proliferation of NPC cells in vitro and vivo.

EGFL6 is an epidermal growth factor‐like protein, it is well known that it plays a series of roles in endothelial cell behaviors and angiogenesis of.^19^, ^20^ Recently, there are some reports prove that EGFL6 involved in the progression of human cancer, such as ovarian cancer,^21^ breast cancer,^22^ meningioma.^9^ However, the relationship between EGFL6 and NPC has not been elucidated clearly.

To further explore the biological function of EGFL6 in NPC, we downregulated the expression of EGFL6in NPC cells. We observed that EGFL6 accelerated the migration of NPC cells (Figure 2D). And when we silenced EGFL6, the cells increased in G0‐G1 phase while they were decreased in S phase (Figure 2E). Further, we stained the transfected NPC cells with proliferation maker KI67, the immunofluorescence staining in shEGFL6 group was much decreased (Figure 2F). All these phenomena indicated that EGFL6 involved in migration and proliferation of NPC in vitro.

AKT is a serine and threonine kinase and facilitates the growth and progression of tumor cells.^23^ The AKT pathway is activated in the migration process of osteosarcoma and gastric cancer.^10^, ^24^ Blocking the AKT signaling resulted in the attenuated capability of migration obviously in breast carcinoma.^25^, ^26^ More interestingly, EGFL6 stimulates activation of AKT signaling in zebrafish.^20^ In our study, we found that p‐AKT was downregulated in the shEGFL6 group (Figure 3A), which means AKT pathway was inhibited when silence EGFL6 in NPC cells. Moreover, loss of EGFL6 and subsequent activation of AKT promoted the migration of NPC cells remarkably (Figure 3B,C). These data suggested that EGFL6 promoted the migration of NPC cells by the activation of AKT pathway.

Furthermore, we found that the growth of NPC in nude mice was reduced in shEGFL6 group (Figure 4), and the migration of NPC in zebrafish was promoted in EGFL6‐overexpression group (Figure 5).

In summary, we provided evidence that EGFL6 was high expression in NPC, and was associated with migration and growth of NPC cells. The results indicated that EGFL6 might play as an important maker in NPC.

## CONFLICT OF INTEREST

The authors have declared that no competing interest exists.
